# Catalytic conversion of carbon dioxide into dimethyl carbonate using reduced copper-cerium oxide catalysts as low as 353 K and 1.3 MPa and the reaction mechanism

**DOI:** 10.3389/fchem.2013.00008

**Published:** 2013-06-26

**Authors:** Seiki Wada, Kazuki Oka, Kentaro Watanabe, Yasuo Izumi

**Affiliations:** Department of Chemistry, Graduate School of Science, Chiba UniversityChiba, Japan

**Keywords:** CO_2_, dimethyl carbonate, cerium oxide, partial reduction, X-ray absorption near-edge structure (XANES), oxygen vacancy, hydrogen subtraction, environmental catalyst

## Abstract

Synthesis of dimethyl carbonate (DMC) from CO_2_ and methanol under milder reaction conditions was performed using reduced cerium oxide catalysts and reduced copper-promoted Ce oxide catalysts. Although the conversion of methanol was low (0.005–0.11%) for 2 h of reaction, DMC was synthesized as low as 353 K and at total pressure of as low as 1.3 MPa using reduced Cu–CeO_2_ catalyst (0.5 wt% of Cu). The apparent activation energy was 120 kJ mol^−1^ and the DMC synthesis rates were proportional to the partial pressure of CO_2_. An optimum amount of Cu addition to CeO_2_ was 0.1 wt% for DMC synthesis under the conditions at 393 K and total pressure of 1.3 MPa for 2 h (conversion of methanol: 0.15%) due to the compromise of two effects of Cu: the activation of H_2_ during reduction prior to the kinetic tests and the block (cover) of the surface active site. The reduction effects in H_2_ were monitored through the reduction of Ce^4+^ sites to Ce^3+^ based on the shoulder peak intensity at 5727 eV in the Ce L_3_-edge X-ray absorption near-edge structure (XANES). The Ce^3+^ content was 10% for reduced CeO_2_ catalyst whereas it increased to 15% for reduced Cu–CeO_2_ catalyst (0.5 wt% of Cu). Moreover, the content of reduced Ce^3+^ sites (10%) associated with the surface O vacancy (defect sites) decreased to 5% under CO_2_ at 290 K for reduced Cu–CeO_2_ catalyst (0.1 wt% of Cu). The adsorption step of CO_2_ on the defect sites might be the key step in DMC synthesis and thus the DMC synthesis rate dependence on the partial pressure of CO_2_ was proportional. Subsequent H atom subtraction steps from methanol at the neighboring surface Lewis base sites should combine two methoxy species to the adsorbed CO_2_ to form DMC, water, and restore the surface O vacancy.

## Introduction

Carbon dioxide is one of major green house gases. The conversion of CO_2_ has been widely investigated to reduce the atmospheric concentration of CO_2_ (Izumi, 2013). In the viewpoint of global warming, fixation methods of CO_2_ and/or converted compounds from CO_2_ are also critical. Transferring captured CO_2_ to the bottom of the sea in a supercritical state is partially in practical use, but it incurs huge investment costs. The conversion of CO_2_ to dimethyl carbonate (DMC) is attractive because DMC can be used as an electrolytic solution of lithium ion battery, methylating reagent, and feedstock for engineering plastics (Ono, 1997).

DMC has been conventionally synthesized starting from phosgene, carbon monoxide, or oxirane, but these materials are toxic and/or explosive. From CO_2_ and methanol/acetals, DMC was synthesized using homogeneous Sn catalysts at 10–30 MPa and 353–453 K (Sakakura et al., 1998, 1999, 2000; Kalhor et al., 2011) and using homogeneous Ni catalysts at 353 K and 1.0 MPa (Shi et al., 2013). Catalyst separation was improved for DMC synthesis using heterogeneous CeO_2_ (Yoshida et al., 2006), ZrO_2_ (Tomishige et al., 1999), solid solution of ZrO_2_ and CeO_2_ (Tomishige et al., 2001; Zhang et al., 2011b), Ga_2_O_3_/Ce_0.6_Zr_0.4_O_2_ (Lee et al., 2011), Ce_*x*_Zr_0.9–*x*_Y_0.1_O_2_ (Zhang et al., 2011a), SnO_2_–ZrO_2_/SiO_2_ (Ballivet-Tkatchenko et al., 2011), Co_1.5_PW_12_O_40_ (Aouissi et al., 2010), H_3_PW_12_O_40_/Ce_*x*_Ti_1–*x*_O_2_ (La et al., 2007), Cu–KF/MgSiO (Li and Zhong, 2003), Cu–Ni–diatomite (Chen et al., 2012), Cu–Ni–graphite (Bian et al., 2009a), Cu–Ni–V_2_O_5_–active carbon (Bian et al., 2009b), and Cu–Ni–V_2_O_5_–SiO_2_ (Wu et al., 2006; Wang et al., 2007) at 353–453 K and 0.1–60 MPa. The conversion of methanol to DMC was as much as 7.9% for 24 h (Zhang et al., 2011b). In the viewpoint of global environment and the reduction of CO_2_, it is desirable to synthesize DMC from CO_2_ under mild reaction conditions.

In this context, the conversion of CO_2_ and methanol to DMC under milder conditions was studied and the mechanism was investigated by X-ray absorption near-edge structure (XANES). Methanol could be synthesized photocatalytically from CO_2_ ((Ahmed et al., 2011, 2012); Morikawa et al. under review). In future, the DMC synthesis reported in this work could be combined with photocatalysis to synthesize DMC from CO_2_ as a single starting material.

## Materials and methods

### Preparation of CeO_2_

Cerium oxide samples were prepared from cerium nitrate hexahydrate (Wako Pure Chemical, >98.0%). It was dissolved in deionized water (<0.06 μS cm^−1^) to make the concentration to 0.2 mol L^−1^. A 5% ammonia aqueous solution (Wako Pure Chemical) was added to the solution to reach the pH 10. Obtained yellow precipitate was filtered using a polytetrafluoroethene-based membrane filter (Omnipore JGWP04700, Millipore) with a pore size of 0.2 μm and washed several times with deionized water. The obtained powder was calcined in air at 673 K for 4 h. Then, the powder was connected to a vacuum system using rotary and diffusion pumps (10^−6^ Pa) and the temperature was elevated at a ramping rate of 5 K min^−1^ from 290 to 673 K and kept at 673 K for 1 h.

A part of freshly-prepared CeO_2_ above was reduced under 25 kPa of hydrogen. The temperature was elevated from 290 to 673 K with the ramping rate of 10 K min^−1^ and maintained at 673 K for 1 h.

### Preparation of Cu–−CeO_2_

3.8–950 mg of copper nitrate trihydrate (Wako Pure Chemical, >99.9%) was dissolved in 10 mL of deionized water. The Cu^2+^ solution was added to 1.0 g of CeO_2_ powder prepared in section Preparation of CeO_2_. Then, 25% of ammonia aqueous solution was added to the suspension until the pH reached 9.5. The mixture was reacted at 290 K for 1 h with magnetically stirred at a rate of 300 rpm. The color of precipitate was yellow, yellow green, and dark brown when the Cu content was 0.1, 1, and 20 wt%, respectively. The precipitate was filtered using JGWP04700 membrane and washed by several times with deionized water. The obtained powder was dried at 353 K for 12 h and denoted Cu–CeO_2_. The Cu–CeO_2_ samples were treated under H_2_ (25 kPa). The temperature was elevated from 290 to 673 K with the ramping rate of 10 K min^−1^, maintained at 673 K for 1 h, and evacuated (10^−6^ Pa) at 673 K for 30 min.

### Characterization

Nitrogen adsorption isotherm measurements were performed at 77 K within the pressure range 1.0–90 kPa in a vacuum system connected to diffusion and rotary pumps (10^−6^ Pa) and equipped with a capacitance manometer (Models CCMT-1000A and GM-2001, ULVAC). The Brunauer-Emmett-Teller (BET) surface area (*S*_BET_) was calculated on the basis of eight-point measurements between 10 and 46 kPa (*P/P*_0_ = 0.10–0.45) on the adsorption isotherm. The sample was evacuated at 423 K for 90 min before the measurements.

The electronic state of cerium in catalysts was investigated by the synchrotron X-ray measurements. The catalyst powder samples were prepared in vacuum (10^−6^ Pa) and transferred directly to a Pyrex glass cell equipped with 50 μm-thick Kapton (Dupont) windows on both sides. The samples in N_2_ (60 kPa) or CO_2_ gas (60 kPa) were sealed with fire and transported to beamline.

Ce L_3_-edge X-ray absorption fine structure (XAFS) spectra were measured at 290 K in a transmission mode in the Photon Factory at the High-Energy Accelerator Research Organization (Tsukuba, Japan) on beamline 9C and also in SPring-8 (Sayo, Japan) on beamline 01B1. The X-ray energy was calibrated at the first intense peak top energy (5731.1 eV) for the Ce L_3_-edge spectrum of CeO_2_. The XAFS data were analyzed with a software package XDAP version 2.2.7 (Vaarkamp et al., 2006).

### DMC synthesis tests

An autoclave (Taiatsu Glass Kogyo, inner volume 120 mL; Model TVS–N2–120) was used for the DMC synthesis tests using CeO_2_ and Cu–CeO_2_ catalysts. The inner space of autoclave was purged with argon gas (>99.998%) at a rate of 300 mL min^−1^. 10 mL of dehydrated methanol (Kanto Chemical, 99.8%) and 100 mg of catalyst were introduced in the autoclave with the flow of Ar, not in contact with air. Next, CO_2_ was flowed at a rate of 300 mL min^−1^ for 15 min. The outlet valve of reactor was closed and the interior pressure was increased to 2.0, 1.0, 0.50, 0.10 MPa at 290 K. Then, the temperature of the reactor was elevated from 290 to 393, 373, or 353 K with the ramping rate of 4 K min^−1^, and maintained at the destination temperature for 2–6 h. As a comparison, 10 mL of dehydrated methanol, 50 mg of catalyst, and 3.6 MPa of CO_2_ were introduced in the autoclave in similar way at 290 K. Then, the temperature of reactor was elevated from 290 to 403 K with the ramping rate of 4 K min^−1^, and maintained at 403 K for 2 h. The reaction suspension was filtered using a JGWP04700 membrane. The filtrate was analyzed by gas chromatograph equipped with frame ionization detector (Model GC-18A, Shimadzu) equipped with a capillary column Ultra ALLOY-5 (Frontier Laboratories; inner diameter 250 μm, length 30 m). The conversion (%) of methanol to DMC was defined as

$$\text{Conversion}(\%)\text{​}=\text{​}\frac{2\times \text{molar amount of DMC formed}}{\text{molar amount of methanol introduced}}\times 100.$$

## Results

### DMC synthesis from CO_2_ and methanol

#### Pretreatment effects in H_2_

In the test under the 2.8 MPa CO_2_ (initial pressure) at 393 K for 6 h, DMC formation rate using incipient CeO_2_ catalyst was 0.44 mmol h^−1^ g^−1^_cat_ and that for reduced CeO_2_ was 0.70 mmol h^−1^ g^−1^_cat_ (Table 1A). Total initial pressure of CO_2_ and methanol was 3.5 MPa at 393 K. By the pretreatment effect in H_2_ at 673 K, the synthesis rate increased by a factor of 1.6 times. The conversion (%) of methanol to DMC was improved to 0.33% (Table 1A).

**Table 1:** **Conditions and results of DMC synthesis from methanol and CO_2_ over Ce oxide and Cu–promoted Ce oxide catalysts^1^**.

					**Initial pressure**				**DMC**		
					**CO_2_**		**Methanol at Reac. T. (MPa)**	**Total at Reac. T. (MPa)**	**Yield (mmol)**	**Synthesis rate (mmol h^−1^ g^−1^_cat_)**	**Conversion to methanol (%)**
**Entry**	**Condition**	**Cu content (wt%)**	**Reaction T. (K)**	**Reaction time (h)**	**at 290 K (MPa)**	**at Reac. T. (MPa)**					
**(A) PRETREATMENT EFFECTS IN H_2_**											
a	Incipient	0	393	6	2.0	2.8	0.64	3.5	0.29	0.44	0.24
b	Reduced	0	393	6	2.0	2.8	0.64	3.5	0.41	0.70	0.33
**(B) Cu EFFECTS AT HIGHER PRESSURE**											
c	Reduced	0	393	2	2.0	2.8	0.64	3.5	0.33	1.5	0.27
d	Reduced	0.5	393	2	2.0	2.8	0.64	3.5	0.40	1.8	0.32
**(C) Cu EFFECTS AT LOWER PRESSURE**											
e	Reduced	0	393	2	0.50	0.67	0.64	1.3	0.087	0.41	0.071
f	Reduced	0.1	393	2	0.50	0.67	0.64	1.3	0.19	0.95	0.15
g	Reduced	0.3	393	2	0.50	0.67	0.64	1.3	0.13	0.64	0.10
h	Reduced	0.5	393	2	0.50	0.67	0.64	1.3	0.13	0.60	0.11
i	Reduced	1	393	2	0.50	0.67	0.64	1.3	0.088	0.43	0.071
j	Reduced	5	393	2	0.50	0.67	0.64	1.3	0.079	0.41	0.064
k	Reduced	10	393	2	0.50	0.67	0.64	1.3	0.038	0.19	0.031
l	Reduced	20	393	2	0.50	0.67	0.64	1.3	0.078	0.39	0.063
**(D) REACTION PRESSURE EFFECTS**											
d	Reduced	0.5	393	2	2.0	2.8	0.64	3.5	0.40	1.8	0.32
m	Reduced	0.5	393	2	1.0	1.4	0.64	2.0	0.18	0.79	0.14
h	Reduced	0.5	393	2	0.50	0.67	0.64	1.3	0.13	0.60	0.11
n	Reduced	0.5	393	2	0.10	0.13	0.64	0.77	<0.003	<0.015	<0.002
o	Reduced^2^	0.1	403	2	3.6	5.8	0.84	6.6	0.29	3.1	0.23
p	Reduced^2^	0.5	403	2	3.6	5.8	0.84	6.6	0.22	1.9	0.17
**(E) REACTION TEMPERATURE EFFECTS**											
d	Reduced	0.5	393	2	2.0	2.8	0.64	3.5	0.40	1.8	0.32
q	Reduced	0.5	373	2	2.0	2.6	0.35	2.9	0.056	0.24	0.045
r	Reduced	0.5	353	2	2.0	2.5	0.18	2.7	0.006	0.031	0.005

^1,2^Catalyst charged: 100 mg except for entries o and p (50 mg).

#### Effects of the Cu addition

The DMC synthesis rate using reduced Cu–CeO_2_ (0.5 wt% Cu) was compared to that using reduced CeO_2_ at 393 K for 2 h. The rates were 1.8 and 1.5 mmol h^−1^ g^−1^_cat_, respectively (Table 1B). By the inclusion of 0.5 wt% of Cu in the catalyst, the rate increased by a factor of 1.2 times. The conversion of methanol to DMC was improved to 0.32% (Table 1B).

**Figure 1:**
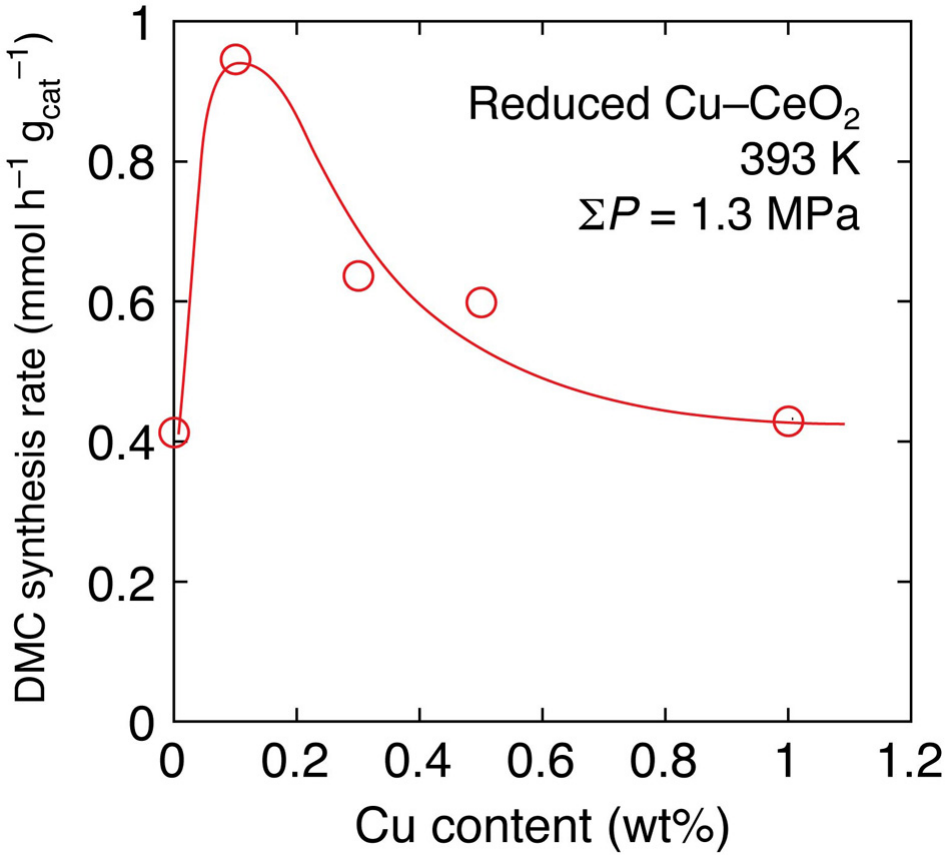
**The dependence of DMC synthesis rates on the copper content in reduced Cu–CeO_2_ catalysts under total initial pressure of 1.3 MPa at 393 K**.

Next, the effects of Cu addition were investigated by progressively changing the Cu content between 0 and 20 wt% under lower initial pressure (0.67 MPa) of CO_2_ at 393 K. By the inclusion of 0.1 wt% of Cu in Cu–CeO_2_ catalyst, the DMC synthesis rate increased 2.3-fold higher: from 0.41 mmol h^−1^ g^−1^_cat_ (reduced CeO_2_) to 0.95 mmol h^−1^ g^−1^_cat_ (Table 1C; Figure 1). However, further increase of Cu content between 0.3 and 1 wt% in Cu–CeO_2_ catalysts was not effective compared to the test results for the Cu–CeO_2_ catalyst, 0.1 wt% of Cu (Table 1C). When the Cu content was between 1 and 20 wt%, the synthesis rates gradually approached to constant, similar to the one for undoped CeO_2_ (0.41 mmol h^−1^ g^−1^_cat_) (Table 1C; Figure 1).

#### Reaction pressure effects

**Figure 2:**
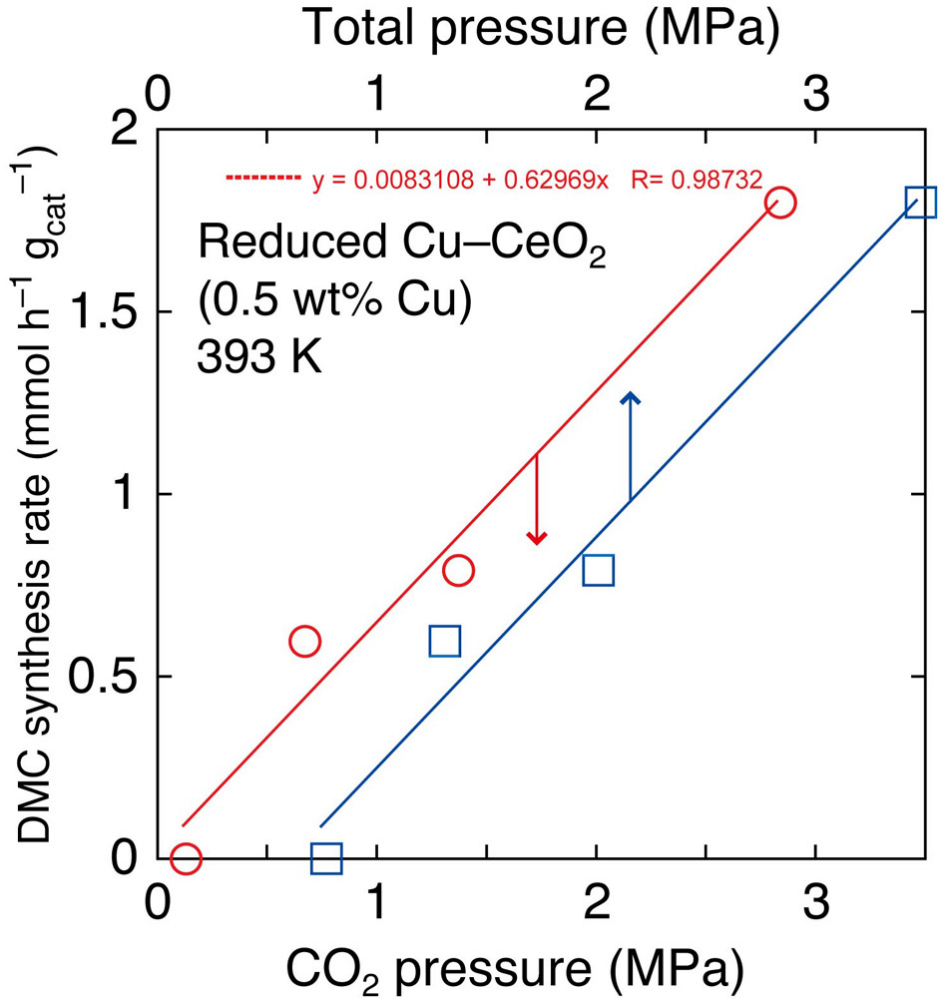
**The dependence of DMC synthesis rates on the partial pressure of CO_2_ and total pressure using Cu–CeO_2_ catalyst (0.5 wt% of Cu) at 393 K**.

In the reaction tests at 393 K using reduced Cu–CeO_2_ catalyst (0.5 wt% Cu), partial pressure of CO_2_ introduced at 290 K was varied between 2.0 and 0.10 MPa. The partial (initial) pressure of CO_2_ increased to between 2.8 and 0.13 MPa at reaction temperature of 393 K (Table 1D). The DMC synthesis rates were plotted as a function of initial pressure of CO_2_ and initial total pressure of CO_2_+ methanol at 393 K (Figure 2). The DMC synthesis was possible under the total pressure of 1.3 MPa, but the amount of produced DMC was below detection limit (3 μmol) under the total pressure of 0.77 MPa at 393 K (Table 1D). The DMC synthesis rates were proportional to partial pressure of CO_2_ (Figure 2).

The reaction pressure effects were also tested under severer reaction conditions: at 403 K and 6.6 MPa using Cu–CeO_2_ catalysts (0.1 and 0.5 wt% Cu) (Table 1o,p). The synthesis rates (3.1–1.9 mmol h^−1^ g^−1^_cat_) were higher by a factor of 3.3–3.2 times compared to corresponding test results at 393 K and 1.3 MPa (Table 1C,D). Thus, major reason of relatively low DMC synthesis rates in this paper was mild reaction conditions at 393–353 K and 3.5–1.3 MPa.

#### Reaction temperature effects

**Figure 3:**
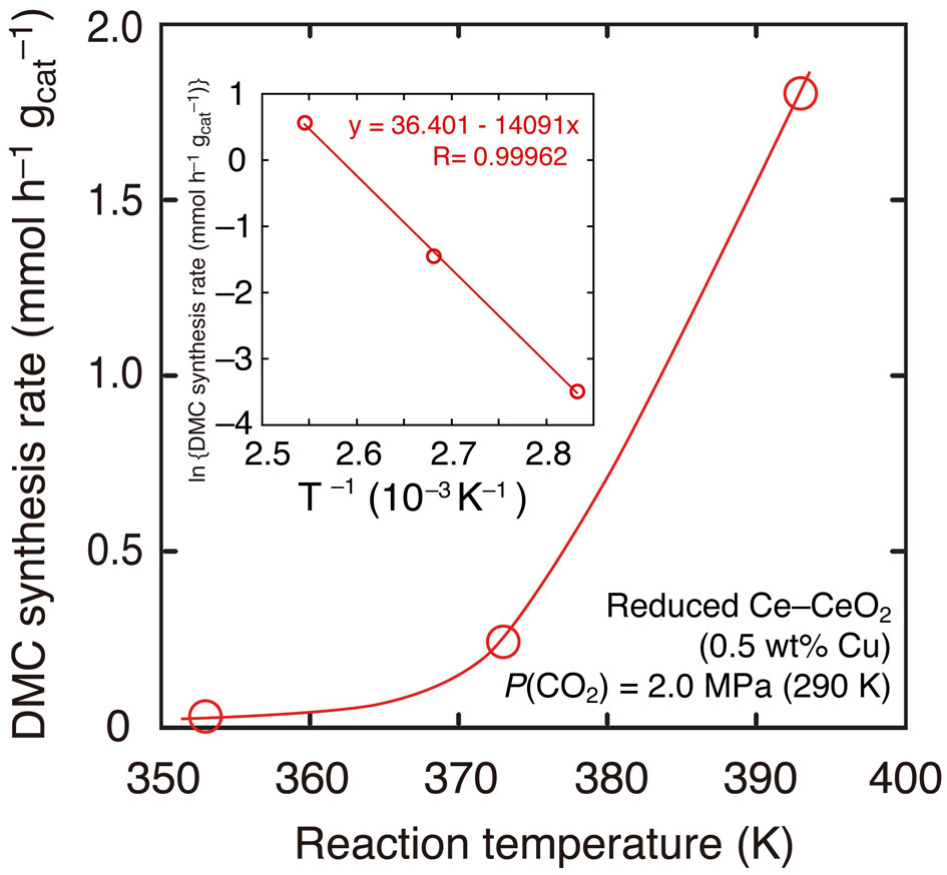
**DMC synthesis rates over Cu–CeO_2_ catalyst (0.5 wt% of Cu) at 393–353 K**. Initial partial pressure of CO_2_ was 2.0 MPa at 290 K. (Inset) The associated Arrhenius plot and the fit to the equation.

Further, the reaction temperature was varied between 393 and 353 K using reduced Cu–CeO_2_ catalyst (0.5 wt% Cu) under the CO_2_ partial pressure of 2.0 MPa introduced at 290 K. The CO_2_ pressure increased to between 2.8 and 2.5 MPa at reaction temperatures of 393–353 K (Table 1E). Under the total pressure of 2.7 MPa, DMC synthesis was possible as low as 353 K: 0.031 mmol h^−1^ g^−1^_cat_ (Table 1E; Figure 3). The apparent activation energy was estimated to 120 kJ mol^−1^ based on the Arrhenius plot (Figure 3, inset).

### BET surface area and Ce L_3_-edge xanes

The BET surface area was 78 and 94 m^2^ g^−1^_cat_ for Cu–CeO_2_ samples consisting of 0.1 and 0.5 wt% Cu, respectively (Table 2).

**Table 2:** **BET surface area of Cu–promoted Ce oxide catalysts**.

**Cu content (wt%)**	***S*_BET_ (m^2^ g^−1^_cat_)**
0.1	78
0.5	94

**Figure 4:**
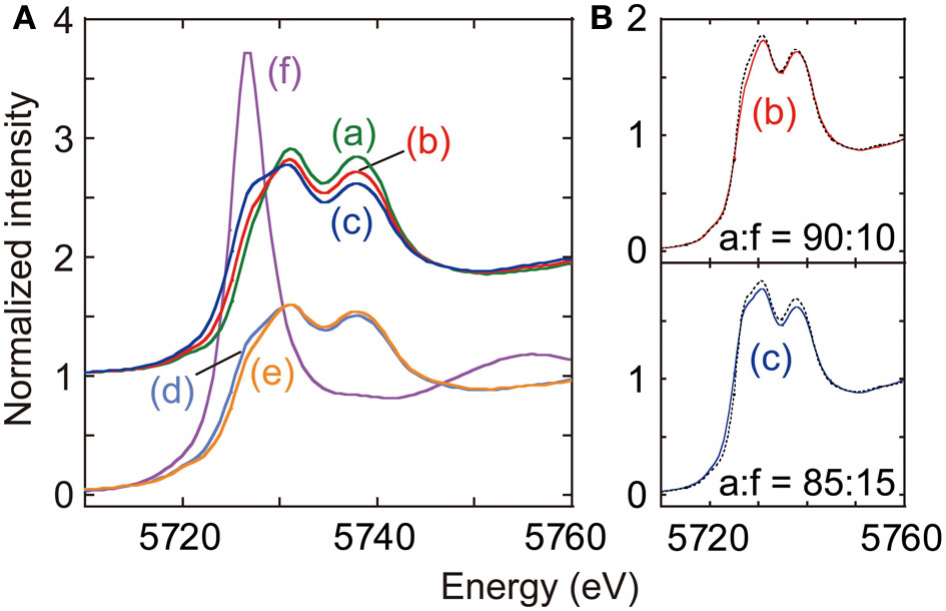
**(A)** Ce L_3_-edge XANES for as-synthesized CeO_2_ (a), CeO_2_ reduced at 673 K (b), Cu–CeO_2_ (0.5 wt% Cu) reduced at 673 K (c), Cu–CeO_2_ (0.1 wt% Cu) under N_2_ after reduction at 673 K (d), Cu–CeO_2_ (0.1 wt% Cu) under CO_2_ after reduction at 673 K (e), and Ce(NO_3_)_3_·6H_2_O (f). **(B)** Fits to spectra (b) and (c) with the combination of spectra (a) and (f). Best-fit results were shown by changing the mixing ratio of data (a) and (f).

Ce L_3_-edge XANES spectra taken for Ce–based catalysts and also standard Ce compounds were depicted in Figure 4. Twin peaks appeared at 5731 and 5738 eV in the XANES spectrum for as-synthesized CeO_2_ (spectrum a), indicating that valence state of Ce^4+^ was predominant (Zhang et al., 2004).

When the CeO_2_ was reduced under hydrogen at 673 K (spectrum b), a shoulder peak gradually grew at 5727 eV, on the lower side of peak at 5731 eV. As an intense whiteline peak appeared at 5726.7 eV in the spectrum for Ce^III^(NO_2_)_3_·6H_2_O (Figure 4A-f), the shoulder peak for spectrum b suggested the partial reduction of initial Ce^4+^ to Ce^3+^. The partial reduction of CeO_2_ is traditionally known to promote electron-donating catalysis, e.g., ammonia synthesis (Izumi et al., 1996; Aika et al., 1997). The spectrum b was fitted with the spectrum a for fresh CeO_2_ and spectrum f for Ce(NO_2_)_3_·6H_2_O by changing the mixing ratio of standard spectra (Izumi and Nagamori, 2000; Izumi et al., 2007). Spectra a and f were used as models of Ce^4+^ and Ce^3+^ states, respectively. The goodness of fit was evaluated based on the residual-factor (*R*_*f*_)

$$R_{f}=\frac{\int {\left|\chi^{\text{sample data}}(k)-\chi^{\text{reference data}}(k)\right|}^{2}\text{d}k}{\int {\left|\chi^{\text{sample data}}(k)\right|}^{2}\text{d}k}$$

The spectrum b was best fitted with the mixing ratio of Ce^4+^:Ce^3+^ = 90:10 (Figure 4B-b).

The shoulder peak at 5727 eV also appeared in the XANES spectrum for reduced Cu–CeO_2_ (0.5 wt% Cu; spectrum c), and the intensity was greater than that in spectrum b for reduced CeO_2_. The spectrum c was also fitted with the spectrum a (Ce^4+^) and spectrum f (Ce^3+^) by changing the mixing ratio. The best fit was realized with the mixing ratio was Ce^4+^:Ce^3+^ = 85:15 (Figure 4B-c).

The Ce L_3_-edge XANES spectrum for reduced Cu–CeO_2_ catalyst (0.1 wt% Cu; spectrum d) was essentially identical with that for reduced CeO_2_ catalyst (spectrum b). The best-fit ratio with the mixing standard spectrum component of Ce^4+^ and Ce^3+^ was 90 and 10%. However, the shoulder peak at 5727 eV in the spectrum significantly weakened when 60 kPa of CO_2_ was introduced to the reduced Cu–CeO_2_ (0.1 wt% Cu) at 290 K (spectrum e). The best-fit ratio with the mixing standard spectrum component of Ce^4+^ and Ce^3+^ was 95 and 5%. The decrease of shoulder peak intensity at 5727 eV suggested re-oxidation of Ce^3+^ to Ce^4+^ by the reaction with CO_2_.

## Discussion

### DMC synthesis under milder conditions

DMC synthesis from CO_2_ and methanol was reported at a synthesis rate of 1.8–5.1 mmol h^−1^ g^−1^_cat_ using CeO_2_ at 403 K and 8.7 MPa for 2–4 h (Yoshida et al., 2006). DMC synthesis rate from CO_2_ and methanol in this work using reduced CeO_2_ at 393 K and 3.5 MPa for 6 h was lower: 0.70 mmol h^−1^ g^−1^_cat_ (Table 1A) due to lower reaction temperature and lower pressure. Because the forward reaction reduces the molar amount of materials in system from three to two and is uphill reaction (Pacheco and Marshall, 1997) (Equation 1), reaction conditions of lower reaction temperature and lower pressure are disadvantageous for the DMC synthesis reaction. The synthesis rate was enhanced by a factor of 1.6 times by the pre-reduction in H_2_ for CeO_2_ (Table 1A).

(1) $${\text{CO}}_{\text{2}}+{\text{2CH}}_{\text{3}}\text{OH}⇄\text{OC}{\left({\text{OCH}}_{\text{3}}\right)}_{2}+{\text{H}}_{\text{2}}\text{O}\Delta G_{\text{r}}=51.0{\text{kJmol}}^{-1}(373\text{K})$$

The disadvantage of moderate reaction conditions was compensated by the Cu addition to CeO_2_ catalysts. At 393 K and 3.5 MPa for 2 h, the DMC synthesis rates increased to 1.8 mmol h^−1^ g^−1^_cat_ by the addition of 0.5 wt% of Cu (Table 1B).

The effects of Cu addition to the DMC synthesis rates were compared at even milder reaction conditions: at 393 K and 1.3 MPa for 2 h (Table 1C). Under the reaction conditions, the 0.1 wt% of Cu was most effective and it promoted the synthesis rate by a factor of 2.3 times (Figure 1). One of the plausible explanations is that the positive effects to induce the Ce^4+^ site reduction to facilitate H_2_ dissociation and spillover on the catalyst surface and negative effects to block (cover) the surface active sites for DMC synthesis, e.g., the adsorption/activation sites for methanol, compromised to make a synthesis rate maximum at the Cu amount of 0.1 wt%.

The maximal DMC synthesis rate using Cu–CeO_2_ catalyst (0.1 wt% Cu) at 393 K and 1.3 MPa was 0.95 mmol h^−1^ g^−1^_cat_ (Table 1f), but the rate at 403 K and 6.6 MPa was quite higher (3.1 mmol h^−1^ g^−1^_cat_), nearly equivalent to those in literature using CeO_2_ (1.8–5.1 mmol h^−1^ g^−1^_cat_) at even severe conditions (403 K and 8.7 MPa) (Yoshida et al., 2006), demonstrating the effects of pre-reduction and/or the Cu addition to CeO_2_ found in this work. The *S*_BET_ values (78–94 m^2^ g^−1^_cat_; Table 2) for Cu–CeO_2_ catalysts (0.1–0.5 wt% Cu) were also similar to those for CeO_2_ catalyst reported (80 m^2^ g^−1^_cat_) (Yoshida et al., 2006).

The effects of ZrO_2_ mixed with CeO_2_ (Tomishige et al., 2001; Zhang et al., 2011b) were also interpreted to enhance the redox chemistry between Ce^4+^ and Ce^3+^. In this sense, the redox of Cu^+^ and Cu^2+^ may enhance the redox between Ce^4+^ and Ce^3+^. We tested Co–CeO_2_ catalyst under the reaction condition of Table 1d. The conversion of methanol to DMC was 0.23% (not listed), slightly inferior to Cu–CeO_2_ catalyst. Furthermore, in our preliminary results, the conversions to DMC using Fe–CeO_2_ and Ni–CeO_2_ catalysts were nearly equivalent to that using Co–CeO_2_ catalyst. Thus, the effects of hydrogen activation and/or redox of added metal (Fe, Co, Ni, or Cu) to CeO_2_ may work in similar way: enhancing effects of mixed metal ions and/or activating effects of hydrogen during pretreatment.

The reactions at lower reaction temperatures were tested (Table 1E). At 2.7 MPa using Cu–CeO_2_ catalyst (0.5 wt% of Cu), DMC was formed at as low as 353 K (Table 1E). The temperature dependence of DMC synthesis rates nicely followed the Arrhenius equation to give the apparent activation energy: 120 kJ mol^−1^ (Figure 3, inset). Similar range of apparent activation energy (107 kJ mol^−1^) was obtained using homogeneous Sn catalysts in the temperature range of 357–403 K (Kalhor et al., 2011). The dependences of DMC synthesis rates on the CO_2_ pressure and total reactant pressure were also investigated at 393 K using Cu–CeO_2_ catalyst (0.5 wt% of Cu; Table 1D). DMC was synthesized as low as 1.3 MPa.

The progress of catalysts to synthesize DMC from CO_2_ is quite fast, especially under relatively mild conditions (see the Introduction section). The Cu–CeO_2_ catalysts in this study are one of the good catalysts to work at relatively mild conditions. The dependence of synthesis rates on pressure and temperature (Table 1) was interpreted based on X-ray spectroscopy in next section by monitoring the oxygen defect sites and Ce^3+^.

### Active sites of DMC synthesis

The dependence of DMC synthesis on the CO_2_ pressure (previous section) was proportional (Figure 2). This fact suggested that the key reaction step of DMC synthesis depended linearly on the CO_2_ concentration.

To provide the insight into the surface reaction mechanism, the electronic state and structure for Ce sites were investigated using Ce L_3_-edge XANES. 10–15% of the Ce^4+^ sites of as-prepared CeO_2_ or Cu–CeO_2_ (0.5 wt% of Cu) were reduced to Ce^3+^ based on the shoulder peak intensity at 5727 eV (Figure 4b,c). Similarly, 10% of the Ce^4+^ sites of as-prepared Cu–CeO_2_ (0.1 wt% of Cu) were reduced to Ce^3+^ under H_2_ at 673 K, but a half of the Ce^3+^ sites were re-oxidized to Ce^4+^ by the introduction of CO_2_ at 290 K (Figure 4d,e). These changes in the XANES spectra suggested the adsorption of CO_2_ at the surface defect sites over the catalyst and the associated, neighboring Ce^3+^ sites to the defects were re-oxidized to Ce^4+^. This reduction and re-oxidation mechanism was already reported on CeO_2_ layers grown over Cu(111) surface (Staudt et al., 2010).

**Figure 5:**
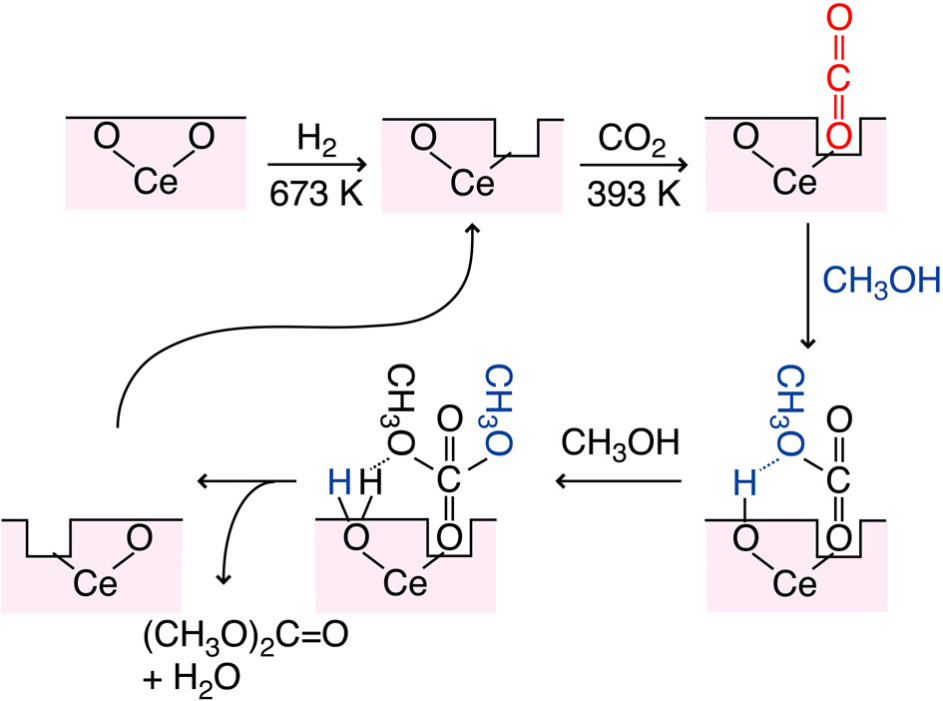
**Proposed reaction mechanism of CO_2_ conversion to DMC using partially-reduced Ce oxide surface**.

The proposed reaction mechanism was shown in Figure 5. Based on the dependence of DMC synthesis rates on the CO_2_ pressure and the change of a shoulder peak at 5727 eV in the Ce L_3_-edge XANES spectra, O vacancy was assumed as defect site and worked to adsorb CO_2_. The population of O vacancy should increase by the reduction in H_2_ and/or by the presence of Cu sites in catalysts. In order to synthesize DMC, surface Lewis base sites are required to subtract H atom from methanol (Figure 5). If each H atom was subtracted at the Lewis base site from two methanol molecules, DMC and water molecules are formed to restore an O vacancy site.

## Conclusions

Reduction of CeO_2_ and Cu–promoted CeO_2_ catalysts in hydrogen at 673 K was effective to enhance the DMC synthesis from CO_2_ and methanol by a factor of 1.6–1.2 times. Added Cu worked cooperatively with CeO_2_ catalysts as it accelerated the partial reduction of Ce^4+^ sites to Ce^3+^. At the same time, doped Cu sites may block surface active sites. As a compromise, the DMC synthesis rate was maximal: 0.95 mmol h^−1^ g^−1^_cat_ at 393 K and 1.3 MPa (total pressure) in 2 h when the Cu amount was 0.1 wt% for reduced Cu–CeO_2_ catalyst. The DMC synthesis was possible at the reaction temperature as low as 353 K (2.7 MPa) using the reduced Cu–CeO_2_ catalyst. The apparent activation energy was calculated to be 120 kJ mol^−1^. Based on the Ce L_3_-edge XANES, 10% of Ce sites were reduced to Ce^3+^ by the reduction in H_2_ for Cu–CeO_2_ (0.1 wt% of Cu) while half of them were re-oxidized to Ce^4+^ by the introduction of CO_2_ at 290 K. A linear rate dependence on CO_2_ pressure and the re-oxidation in CO_2_ suggest that the adsorption of CO_2_ might be the key step in DMC synthesis. H subtraction from methanol needs to occur at the neighboring sites of adsorbed CO_2_. Two methoxy groups and adsorbed CO_2_ combine then to form DMC and water and restores surface O vacancy (defect site).

### Conflict of interest statement

The authors declare that the research was conducted in the absence of any commercial or financial relationships that could be construed as a potential conflict of interest.
